# African-American Fathers’ Perspectives on Their Children’s Health Education: A Qualitative, Exploratory Study

**DOI:** 10.3389/fpubh.2014.00276

**Published:** 2014-12-08

**Authors:** Mary Odum, Matthew Lee Smith, E. Lisako J. McKyer

**Affiliations:** ^1^Department of Health and Human Performance, Texas State University, San Marcos, TX, USA; ^2^Department of Health and Kinesiology, Texas A&M University, College Station, TX, USA; ^3^Department of Health Promotion and Behavior, College of Public Health, The University of Georgia, Athens, GA, USA; ^4^Department of Health Promotion and Community Health Sciences, School of Rural Public Health, Texas A&M Health Science Center, College Station, TX, USA

**Keywords:** African-American fathers, paternal perspectives, child health, health education, qualitative

## Abstract

**Purpose:** To investigate African-American fathers’ (AAF) perceptions regarding the applicability and need for their involvement as a health connection for their children and describe how participating fathers’ behavior was affected by their attitudes, knowledge, and perceptions of their influence on their children’s health.

**Methods:** This exploratory study gathered data via semi-structured focus groups (*n* = 3) and thematically analyzed it utilizing a grounded theory approach. Participants included AAF (*n* = 20) with a mean age of 37 years (SD 11.79), with at least one child between 6 and 18 years old.

**Results:** Four major themes were revealed: (1) appropriate health education for participants’ children (should first and foremost be delivered by parents); (2) participants’ paternal health-related guidance approach (reactive, rather than proactive); (3) participants’ perceived influences on health-related communication with their children (gender roles, efficacy constraints); and (4) paternal definitions of health (most often associated with diet).

**Conclusion:** Understanding AAFs’ perceived and desired role in their children’s health edification can inform initiatives that actively engage these men, and nurture their level of involvement, to promote positive health behaviors among their children; this is necessary to realize their potential to actively improve the health of their children, families, and communities.

## Introduction

African-American mothers’ impact on their children’s health is well-documented; in contrast, paternal impact on child health is largely unexplored (1). While studies examining African-American fathers’ (AAF) influences on child health do exist (2–5), many are limited in scope. Additionally, data about AAF influence on their children’s health are often gathered from mothers, which may have a weaker predictive validity than paternal reports (6). Specifically, AAF own perspectives about their role and influence on their children’s health is largely absent from health literature.

Limited information about paternal influence is attributable, in part, to erroneous perceptions that AAF are not involved (or do not wish to be involved) in their children’s lives (7–9). Such assumptions about AAF involvement may perpetuate stereotypes or foster misconceptions about their paternal guidance, thus hindering opportunities for including their valuable insight in research and child health interventions. Indeed, more research is needed about paternal involvement, namely from fathers’ own perspectives (1).

African-American fathers’ involvement in their children’s health can promote positive outcomes. For example, Boyd, Ashcraft, and Belgrave found that AAF involvement in their daughters’ lives increased their daughters’ self-efficacy related to drug refusal (3), and Peterson reported AAF positively impacted their daughters’ sexual-risk-taking behavior (4). Additionally, positive AAF–son sexual-risk communication is associated with son’s reporting less difficulty in discussing sexual topics with romantic partners (5). Given that sexually transmitted infections and unintended pregnancy remain high within the African-American community (10), such positive paternal influence underscores the need to further explore how AAF impact their children’s health to inform health promotion research and practice.

Studies investigating AAFs’ perceived role in their children’s rearing reveal the desire to be involved as caregivers and health educators (7, 11, 12). However, AAF are faced with numerous barriers, including mothers limiting the father–child relationships, especially when fathers do not live with their children. For example, one investigation revealed mothers running interference between non-resident fathers and their children, which the fathers viewed as attempts to thwart the father–child relationship (13). In this case, both maternal interference and non-residency hindered paternal involvement, not lack of paternal desire.

Non-residency, by itself, is a barrier to the AAF–child relationship (14) AAF living apart from their children reported difficulties maintaining relationships and open communication with their children (15) Additionally, non-resident AAF have expressed dissatisfaction with their perceived low involvement with their children (16), further highlighting their desire to be involved fathers.

The authors agree with Tucker that it is “important to acknowledge and validate the various ways that African-American males are involved in mentoring and childrearing” (80, p. 185), especially because this population is often overlooked by researchers, the media, and society at large. Much of what is accepted about AAF involvement in their children’s lives is reported by maternal figures, begging the question, what do fathers, *themselves*, view as their role in their children’s lives? Specifically, this study focuses on AAF perceived roles in their children’s health education.

The current study focused on four research questions: (1) How do fathers perceive their role as health liaisons for their children? (2) Who do fathers believe should be responsible for influencing their children’s health behaviors? (3) What health topics to fathers believe to be important for their children?, and (4) What perceived barriers hinder fathers’ role as health liaisons for their children?

## Materials and Methods

### Design

The current study comprised Phase 1 of a four-phase exploratory study designed to identify AAF perceptions of their influence on their children’s health (17). The ultimate objective of this larger study was to collect quantitative data from a geographically diverse group of AAF in the United States. Phase 1 (the current study) was the qualitative phase of data collection intended to gather subjective data from AAF to inform the creation of a survey instrument. Phase 2 was the instrument development phase, which included identifying existing measures, drafting instrument items, and soliciting expert opinions about the questionnaire. Phase 3 was the pilot testing phase where data were collected from approximately 50 AAF in one Southwestern city. Phase 4 will be the instrument refinement phase (psychometric property testing), which will be followed by grand-scale survey. Specifically, the current study used focus groups to examine: (1) AAFs’ perceptions of the applicability and need for their involvement as health liaisons for their children; and (2) how participating fathers’ perceptions, attitudes, and knowledge of their influence on their children’s health affected their (paternal) behavior.

### Procedures

Purposeful recruitment involved flyers, direct approach, and snowballing techniques. First, flyers and direct approach were utilized in local establishments such as churches, barber shops, and grocery stores. Study information was made available to potential participants; those who expressed interest and provided consent were later contacted by phone to schedule attendance at a focus group. Second, because locating willing participants was initially difficult, snowball recruitment was added, with a $10 incentive offered to participants for referrals.

Inclusion criteria included: (1) be an AAF; (2) speak English; (3) be at least 18 years old; and (4) have a biological child between 6 and 18 years old. Twenty fathers participated; the mean age was 37 years (Table 1). The majority was employed (65%), married (55%), had attained at least a high school degree (75%), and had a child living at home full-time (55%).

**Table 1:** **Participant characteristics**.

Participants (*n* = 20)
Age in years (SD)	37 (11.79)
Employment status (%)	
Employed full- or part-time	65
Marital status (%)	
Married	55
Single	30
Divorced	15
Education (%)	
High school graduate	30
Some college	25
College graduate	20
Residency status (%)	
Child living in home full-time	55
Child living in home part-time	20

We developed and piloted a focus group interview guide (Table 2), which included semi-structured, open-ended questions designed to elicit participant perceptions about the best health information for their children and their perceived role (if any) in their children’s health.

**Table 2:** **Select questions from semi-structured interview guide**.

What health topics do you talk about with your child(ren)?
1.1 What made you bring up those topics?
Give me an example of a health discussion that you spoke to your kids about that took place between you and your child(ren)
Do you think your child has learned any health behaviors from watching you?
Are there certain topics that only you should discuss with your child?
Is there any topic that you anticipate talking to your child about that you have not yet?
Who do you think influences your child as far as health behavior goes?
Who do you think should be responsible for talking to your kids about health topics?

Questions were created using previously-identified paternal influences on children’s health-related behavior. Identical protocols were used for all focus groups.

Our primary lesson learned from pilot testing the interview guide is that we need to enhance the facilitator training to improve the use of probes during the focus group. When reviewing the verbatim transcripts, we identified opportunities for probing, which were not acted upon. We believe this may have limited our understanding about certain aspects of these fathers’ perceptions and their perceived role in their child’s health. Adding more probes to the interview guide might facilitate an increased use of probing.

Additionally, if we were going to do these focus groups again, we would also ask more about technology and peer influence. We touched on these things, but in recent years, these issues have become more pervasive and recognized as a health influence. We would also expand our inclusion criteria to gain information from non-biological fathers and other AA male caregivers/role-models.

Three focus groups were conducted in a rural Southwestern location, moderated by a trained AAF from the local community. All three focus groups were conducted in a local neighborhood recreation center. The study moderator utilized the interview guide to lead the groups’ discussion about: (1) health topics discussed with children; (2) motivation for discussing these health topics; (3) perceived parties responsible for discussing health with children (e.g., the school, parents); (4) perceived influencers on children’s health; and, (5) perceived barriers for discussing health topics with children. Additionally, the moderator employed contextual probes to invite expanded or clarification responses, and one author was present to explain study purpose and procedures, and take field notes.

Focus groups lasted approximately 1 h, included between four and eight participants, and were audio recorded. Recordings were transcribed verbatim and supplemented with field notes taken by the moderator and the attending investigator. Participants provided consent for participation and audio recording, completed a demographic questionnaire, and received a $25 gift card and a meal at the time of the focus group. All study procedures were approved by the Texas A&M University Institutional Review Board.

### Data analysis

Coding and theme development were conducted via ATLAS.ti (version 5.2) (18). First, data were coded line by line using Strauss and Corbin’s grounded theory approach (19). Second, potential relationships between codes were examined and initial themes developed. A second round of coding was conducted, using the initial themes as a guide, resulting in the four major themes presented: these four themes were further explicated by defining their scope with subthemes and corresponding participant quotes.

## Results

Four major themes were revealed, which included AAFs’ perceptions about: (1) appropriate health education for their children; (2) their health-related guidance responsibilities; (3) constraints on parent–child communication; and (4) health topics perceived to be relevant to their children (Table 3).

**Table 3:** **Themes, subthemes, and subtheme code frequencies**.

	# codes
Theme #1: appropriate health education for their children	
Health education should come from parents	69
Health information from other sources should be distrusted (including medical establishment)	60
Health information taught to children was largely based in personal/familial experience	157
Theme #2: parental guidance approach	
Conscious of setting positive, healthy examples for their children	49
Guidance approach strongly rooted in personal experience	85
Guidance related to health education was reactive in nature	65
Health behaviors were reinforced through discipline	10
Paternal role viewed as both protector and teacher	59
Guidance approach influenced by religious beliefs	22
Theme #3: constraints on parent-child health communication	
Perceived gender roles: only men can talk to boys and women can talk to girls about sex and puberty (unless same-sex parent is unavailable)	110
Need to be subversive/sneaky: fathers worried about being hypocrites in their own diet, drinking, or smoking habits	39
Low paternal self-efficacy about ability to deliver age-appropriate health education appropriate for their children	34
Children’s unhealthy food preferences seemed unbeatable	18
Schedules and geographic distance: hectic schedules and non-residency left little time to talk with children	17
Theme #4: most relevant/important health topic for their children	
Diet	57
Physical activity	27
Sexual activity	21
Alcohol, tobacco, and other drugs	19
Relationships	14
Violence	13
Personal Hygiene	10
Obesity	5

### Appropriate health education for children (theme #1)

Three subthemes were embedded within the first theme. First, AAF believed that health education was first and foremost a parental responsibility. Participating fathers felt that parents should teach their children about health before other people attempted to teach them:

*So I feel like, yes, we should discuss certain topics, but we should be the first one and not allow them to hear from somebody else before they hear it from us*.

One rationale provided for parents teaching their children about health issues was the belief that parents put their children’s welfare first:

*There are certain things that only you would teach your children […] because they need to hear it from you first because you gonna have their best interest at heart. Whereas, [when] they teach it in the school, they gonna teach it generalized*.

While the majority of fathers felt strongly that *only* parents should teach their children about health, some believed that it was acceptable for their children to learn about health topics from other sources, *but only if* parents were unavailable to do so.

Second, participants distrusted health information from other sources, including the medical establishment. Fathers viewed providing health education to their children as a paternal responsibility to ensure a proper edification:

*There’s people out there, if you don’t want to teach your kid, but they don’t know, ‘cause they could be teaching them wrong. So if you don’t teach them right at home, then someone else comes along and teaches something wrong*.

Third, familial experiences were highly influential. Participating AAF used family members’ illnesses as a learning opportunity for their children:

Because my dad right now, ‘cause of not taking care of himself, he’s had 2, 3, 4 toes amputated because of being a diabetic and he’s not a big man at all. He’s very small. He’s smoked all his life, so I stressed those issues with my boys, “look at your granddad.”

### Parental guidance approach (theme #2)

Participating fathers had much to say about their parental guidance responsibilities: responses are grouped into seven categories.

#### Role modeling

First, participants were conscientious about setting positive, healthy examples for their children, and believed that children watch and imitate parental behavior:

*Whatever you do, your kids pick up on, and it be passed down. My attitude toward, if I had uh only eat what I wanna eat, my son would pick up [it].My wife says,” I’m cutting you off steak.” She says, “no more red meat” and they [the children] hear me say, “I work every day and if I want steak, I’m gonna have steak!” And they pick that up. […] But yeah, your kids, everything you do they watch you [do], and it affect them more than what you think*.

#### Personal experiences

Second, parental guidance style was rooted in fathers’ personal experiences. Fathers believed that it was important to be honest about their own (past) health-related behaviors when communicating with their children:

*I discuss [my past experiences] with my daughter ‘cause…the path I went, I can say I regret it, and I don’t want her to go near those paths. I had fun, but I try to tell her…I’ve been there. The things I tell her [are] to stop her going through that tribulation, and [through] trials worse than what I went through. If I had listened, like she should listen, like I tell her to, it’d save a whole lot of things…later in life…and I try to tell her this*.

A few fathers admitted hiding unhealthy behaviors from their children, or telling their children not to follow their unhealthy example:

I try to tell them to eat right and exercise and do the things they need to do so you wouldn’t, you know, they health wouldn’t be like mine. I always tell them, “you know, don’t be like me, be better. So take care of yourself.”

#### Reactive health education

Third, most fathers reported approaching health education in a reactive manner; they often waited until an issue arose to discuss it with their children rather than discussing prevention methods proactively:

*I wait ‘cause…I really wouldn’t know how to ‘splain [it] to him unless he’d been in the situation before*.

#### Discipline as behavioral reinforcement

Fourth, fathers believed that they could reinforce their children’s behaviors – including health behaviors – with discipline, although there was a consensus that it was harder to discipline children “nowadays.” Discipline was further described as teaching children between “right and wrong” so that children could learn how to make “right” decisions:

*If they do something wrong, I tell ‘em, ‘keep doin’ it [and] I’m gonna make you go sit in the corner for 5 minutes; you gonna lose your TV privileges; you can’t play with your toys; you keep on, [and] we[‘re]gonna do some pushups. ‘Cause trust me, after a while you gonna be tired of doin’ that*.

*I let ‘em make them mistakes and then explain to ‘em, ‘this is your consequences’. You can’t whoop a child for everything they do. You gotta teach ‘em right and wrong*.

#### Fathers as protectors and teachers

Fifth, participating AAF perceived themselves as both protectors and teachers. They explained that a father’s unique role is to be as involved as possible with their children, to encourage them to make healthy decisions:

*So I think, as fathers, today, with all that comes at our children, we’re gonna have to armor up and realize that all of our children[‘s] health issues, the way I’m looking at it – I’m speaking for myself – that it’s imperative that we try to be as involved in it as much as possible*.

#### Religion and spirituality

Sixth, parental guidance styles were influenced by religious beliefs. God and spirituality were identified as important health matters, upon which life decisions were based. The belief that God should be “put first in life” and everything else (including health issues) would “work out correctly” was evident among multiple participants:

*Try to keep God in your life, because that’s what it’s all about. Put him first and everything else will work out correctly*.

*I really talk to them about their spiritual health, to be honest with you. You know, more than anything, because like I said, we have choices*.

### Constraints on parent–child health communication (theme #3)

Multiple constraints on health communication with their children were noted, including: perceived gender roles; child age; scheduling and geographical difficulties; child food preferences; and, fathers’ need to be sneaky and/or subversive.

Participants believed that mothers had more responsibility to ensure their children consumed a healthy diet because women were perceived to have more control over food purchases and meal preparation for the household. An even stronger theme related to gender specific sex education emerged, the idea that men should talk to boys and women should talk to girls about sex, puberty, and the child–adult transition:

*Because a woman can’t teach no boy how to be a man. It’s impossible. She can teach him morals, but she can’t teach them how to be a man.” Let’s just say this – get you a razor and shave. That’s what a woman’ll tell you. But she won’t tell you if you go up against the grain, it’s gonna bump you up. You see what I’m saying? A woman can’t teach no boy how to be a man. Just like no man, unless he’s a “sheman,” could try to teach a girl how to be a woman. Now you can talk to ‘em and teach them morals to the best of your dealing with a female and what you’ve seen and nine times out of ten what you gonna do is relate her to your mother, your grandmother or your wife. And that’s the realness of it. You gonna relate them, because what you’ve see*.

*I think that we go back old school women should talk to women, the girls. And the men should talk to the boys. Because there are some things that woman can tell them and a girl that a man can’t*.

*So mothers should talk to their daughters; that’s that girl talk. They should sit down and mother talk to her daughter, you know, find out what’s goin’ on and any kinda way she can tell her about her hygiene you need to do this and that*.

Participants viewed sex and puberty as very important health topics, but almost all men felt extremely uncomfortable with and unknowledgeable about menstruation issues, a topic that was widely discussed during the focus groups. The groups’ consensus was that *sometimes* fathers can teach daughters and mothers can teach sons, *but only when* the same-sex parent is unavailable:

*You might have a daughter, or something, and their mother, there’s not a mother around and that’s the time when the daughter may come sit down and want to talk to you and ask you all these questions and you gonna have to be ready to talk to them about this type of stuff. I was just sitting over there thinking about this and there’s a lot of ‘em that don’t have mothers around but they fathers’ around. So you just gotta be prepared*.

*See that’s like my niece, and my nephew, well, there wasn’t a Daddy around, so she had to talk to him about you know, that type of stuff and ‘cause you know, he didn’t there wasn’t a man around to talk to him about then. So she had to talk to him about you know, that sexually transmitted disease stuff, you know and stuff like that*.

Participants believed that age-appropriate health information was important, but were unsure about their ability to adequately present such information to their children. They reported that hectic schedules left little time to talk with children, especially for non-resident fathers (whose children did not live with them, or lived with them part-time). Father–child health communication was further inhibited by fathers’ perceived need to conceal their own, current unhealthy behaviors, especially behaviors related to dietary habits, alcohol use, and tobacco use.

### Relevant health topics for children (theme #4)

Participants identified eight health topics relevant to – and important for – their children; in order of most frequently mentioned: diet, physical activity, sexual activity, alcohol, tobacco, and other drugs (ATOD), relationships, violence, personal hygiene, and obesity (Table 3).

#### Diet

Diet was the most frequently identified health topic participants deemed relevant for their children. While diet was synonymous with health for some, the definition of health was generally multi-faceted. The majority of participants perceived healthiness to be more complex than merely maintaining a healthy weight; however, diet was the most commonly mentioned element. Participants discussed their attempts to improve their own dietary habits, and those of their children, more frequently than any other health topic:

*I try to talk to my kids about eating vegetables and having a more balanced diet, but the way it is with me and my wife working, it’s hard to do that so we eat [at] a lot of fast food places a lot of times. So I try to get them to at least sit down and eat a decent meal, and cook a decent meal. So I really try to talk to them about sitting down and stop running so much and then try to eat a balanced, a more balanced diet*.

*We stress um, eating proper and we try to uh, cut back on the sweets, we think that going to the dentist, you know cavities, so we try to cut back on the sweets, the sodas, which is kinda hard, especially in this day and this day and time. And I love Dr. Pepper so it’s kinda hard to keep the sodas out of the house, but that’s what we stress*.

#### Physical activity

The second most commonly identified component was physical activity. Physical activity was considered important for offsetting poor nutrition (“bad diets”). Fathers reported encouraging their children to be active and exercising with their children:

*I try to, try to stay in shape. Try to stay in shape. I try to play with ‘em a lot, run around, you know what I’m sayin’. Just be active. I box with him though*.

I tell mine, the girls do run around and stuff, take ‘em outside so they can run around, keep their blood pumping and everything, keeping in shape.” The one-year-old, he big so I make sure I keep, you know what I’m saying, some healthy stuff, ‘cause when he get older, I’ll push him into football. And my girl, no telling what she gonna do, but she when she do come over here, I talk to all 3 of them, even though the youngest one don’t understand, I still hold ‘em conversation with ‘em and tell ‘em what’s up, you know what I’m sayin’?

*My thing is this, you can eat certain things as long as you are capable of burning it off*.

#### Sexual activity

The third most common health topic was sexual health. AAF agreed that their children should be educated about sexuality, but perceptions about acceptable sexual behavior differed to some degree:

*Number one thing that I discuss with them, even though I got a little girl and my wife talk to ‘em and I talk to ‘em, is sex. If you gonna do anything, make sure whoever you doin’ something with is always strapped up*.

*I want him to be smart and protect himself […] especially his sexual self, that’s something that I think a father should do. We should be okay about talking about that, just don’t want the wrong people talking about those things*.

*They [schools] teach you [to] use condoms; spiritually that’s unhealthy*.

#### Alcohol, tobacco, and other drugs

Alcohol, tobacco, and other drugs was the fourth most frequently mentioned health topic; tobacco and “drugs” were mentioned more frequently than alcohol. AAF wanted their children to avoid ATOD use, usually because they have used or were currently using ATOD in their lives:

*My little boy came in the house, like “Dad, what’s he smokin’? It smells funny…“ and I said “Please don’t do that when you get older. I said he’s smoking illegal drugs, blah, blah, blah, stuff like that. You know, stay away from that bad stuff.” He’s like, “I just want to know what he smoking. I know you smokin’ cigarettes and Black and Mild, but they don’t smell like that.” You know, I just had to let him know what it was*.

I talk to him about the same thing – not smoking too, ‘cause I smoke myself and I’m bad smoking myself, but I don’t wanna him pickup my habits and everything.’

*‘My main concern like, would be pertainable to drugs, you see what I’m saying? And that’s basically what they promote is tobacco, alcohol and, and, and drugs*.

#### Relationships

Relationships (both romantic and platonic), were the fifth most common health topic mentioned. Participants discussed wanting to protect their daughters from “bad boys” and to increase leadership traits and self-esteem in their sons. These relationship topics were deemed very important to the overall health and happiness of their children:

*I talk to my sons about how to be happy; all mind and be invisible, and don’t be a follower. And how to uh, uh, do things that’s gonna make them a good person in life and in, in and take care of they own*.

*You can get a man anytime, get your values and you like to spend money, you like to have things. Get you an education; get you a good job and all that will come in hand. That’s sure*.

#### Violence

Violence was only mentioned by one focus group; topics concerned gangs, fights, and murder:

*If they get into a situation, be a bigger man or be a bigger woman and walk away from it, before you end up…you gonna hurt this person and hurt their family, but you hurt yourself, too, being away from your people. And the way things are going now, 9 times out of 10, they gonna kill you, too*.

#### Personal hygiene

Personal hygiene was identified as an important health topic. Specifically, showering, hand washing, brushing teeth, avoiding germs, and shaving were addressed:

*I try to make sure she [daughter] do her hygiene every morning and she wash her hands every time she in the bathroom. And I try to tell her just to take care of herself really*.

#### Obesity

Obesity was also identified as a relevant health topic, although participants were uncertain about what, exactly, constituted obesity. They knew being overweight was not healthy for their children, and believed that it was important to help their children avoid becoming obese:

*[We talk] a lot about fast foods, they’re putting weight on and everything, so you try to talk to them about that and try to cook at home a lot more*.

*I guess the main thing that I see, you see on TV or in the news that they always talking about America obesity and about being big and large and they always showing big people and I guess that’s one of the things that, I’m for people having meat on their bones, but at the same time, you got to stay away from just be overweight*.

## Discussion

Four major themes emerged, revealing AAFs’ perceptions about:(1) appropriate health education for their children (should first and foremost be delivered by parents); (2) their paternal health-related guidance approach (reactive, rather than proactive); (3) influences on health-related communication with their children (gender roles, efficacy constraints); and (4) paternal definitions of health (largely synonymous with diet).

The majority of participants felt strongly that health education should come only from parents; however, AAFs’ reported health education approaches were largely reactive (a few did report proactive approaches). This may be problematic, as preventive, proactive approaches to health are more conducive to achieving optimal health. Additionally, fathers distrusted treatment and information from the medical field, limiting their health care resources, including health information. The reported reliance on familial health experiences to guide family health messages narrowed the scope of health edification (education). Therefore, one challenge for the health field is to engage fathers to be more proactive, without infringing upon their perceived health education role.

The second theme revealed that fathers desired to be “good” examples for their children and were conscientious about their role-modeling behaviors, a finding consistent with previous literature (7). Some fathers even reported hiding their unhealthy behaviors from their children, illustrating their awareness of their influence on their children’s behaviors. However, being aware that their behaviors are bad enough to hide from their children raises another set of issues; namely, the need for an intervention to protect the fathers’ health. Also, children might not be as naïve as parents think; they might be aware of unhealthy behaviors their fathers believe that they are hiding. Health promotion efforts need to reinforce fathers’ perceived responsibilities, devising ways to assist fathers in their health guidance endeavors; namely, bringing prevention to the forefront.

Religious beliefs also permeated fathers’ perceived guidance roles related to health, a finding consistent with the African-American faith community (20). Such beliefs may contribute to the reactive health education approach (“…Try to keep God in your life, because that is what it is all about. Put him first and everything else will work out correctly”). Working with such beliefs, rather than against them, may facilitate a positive relationship with these fathers, as previous studies suggest (21).

Parent–child communication was constrained by gender roles, most notably when discussing sexuality. The prevailing consensus was that mothers should talk to daughters, and fathers should talk to sons about sexuality, unless the same-sex parent was unavailable; this restriction limits the opposite-sex parents’ unique and powerful influence. Fathers play an important role in the sexual health of both their sons and daughters. For example, one study found that African-American female college students who had greater communication about sexual risks with their fathers had more positive attitudes toward condom use in the near future, while the college age sons who had greater communication about sexual risks with their fathers reported less difficulty in discussing sexual topics with their partners (5).

### Limitations

This sample only represents a small region in a southwestern location; therefore, the results are not necessarily generalizable to other AAF populations. Additionally, we did not collect data about nationality or birthplace: not tracking this information may decrease the generalizability of findings and serve as a way we could alter the overall study in its future phases. Although information was gathered about the participants’ relationship with their children’s mothers, and their residency status, father–child relationship was not captured directly. This may have influenced the way responses were given by the fathers, thus influencing data interpretation. Additionally, the focus group moderator was a lay-trained AAF, which was positive in that participants related to him and trusted him, but was limiting because he was not a qualitative researcher. Despite training provided by the research team, the moderator employed limited probes during the focus groups to tease out the nuances from participants’ answers. Lastly, this study only included biological fathers; non-biological father figures may provide an expanded perspective.

### Implications

In health promotion, it is important to be aware of perceived gender restrictions and work to understand why these beliefs are so prevalent and how to support parents’ sex and heath communication. Additionally, health promotion efforts may be able to assist fathers’ self-efficacy related to education delivery; as fathers were unsure of their ability to deliver age-appropriate health information. Given fathers’ desire to be good role models, perception as the best person to deliver health education, and apparent love for their children, customized educational materials may be very helpful indeed. As this population has been historically ignored in the literature, this is an important step to reaching fathers, and their children.

An active engagement of AAF in health promotion initiatives related to their children’s health is warranted, especially given the fact that AAF believe that health education should come from parents, first. As such, efforts may utilize resources like the *Starting the Conversation* (22, 23) series or materials offered by organizations like *Head Start* (24), which provides tools and resources that can be used in community settings to initiate dialog about topics including physical activity, nutrition, and tobacco. Health education efforts should focus on nurturing AAF level of involvement in their children’s health, through the promotion of proactive, age-appropriate health communication with their children, in accordance with positive parenting and positive parent–child communication *Healthy People 2020* objectives (25). For example, health educators may leverage existing grand-scale efforts like Fatherhood Buzz (26, 27) or the Fatherhood Initiative (28), which provide educational opportunities for fathers and link them to community resources to strengthen parenting skills and families. Additionally, supporting AAF self-efficacy development related to providing health education to their children is recommended; customized, father–child health education delivery programs may aid this endeavor.

## Conclusion

This exploratory study found AAF report engagement in their children’s health edification, strongly believed that parents should be the health educators for their children, desired to set good examples for their children to follow, and connected religious beliefs to health behaviors as a pedagogical tool when instructing their children. However, these fathers expressed several barriers to communicating with their children about health, including gender-role beliefs that inhibited cross-gender communication regarding sexuality. Implications for research include identifying how to best address the perceived communication barriers to foster AAFs’ potential to positively impact the health of their children, family, and community.

## Conflict of Interest Statement

The authors declare that the research was conducted in the absence of any commercial or financial relationships that could be construed as a potential conflict of interest.
